# Translational research in testicular migration

**DOI:** 10.1590/S1677-5538.IBJU.2021.99.03.1

**Published:** 2020-11-18

**Authors:** Luciano A. Favorito

**Affiliations:** 1 Universidade do Estado de Rio de Janeiro Unidade de Pesquisa Urogenital Rio de JaneiroRJ Brasil Unidade de Pesquisa Urogenital - Universidade do Estado de Rio de Janeiro - Uerj, Rio de Janeiro, RJ, Brasil; 2 Hospital Federal da Lagoa Rio de JaneiroRJ Brasil Serviço de Urologia, Hospital Federal da Lagoa, Rio de Janeiro, RJ, Brasil

## COMMENT

One of the most important research studies of the Urogenital Research Unit is about Testicular Migration. In the interesting paper of Logsdon and Collegues of our research group (1) we can observe a review about the role of the abdominal wall in testicular migration process during the human fetal period. Testicular descent is a complex process of relevant importance for the comprehension of cryptorchidism. There are several anatomic and hormonal factors involved in the testicular migration process (2–4). In present paper (the cover of this edition of Int Braz J Urol) the authors concluded that the abdominal pressure wound is an auxiliary force in testicular migration. Patients with abdominal wall defects are associated with undescendend testis in more than 30% of the cases probably due to mechanical factors; the Prune Belly Syndrome has anatomical changes in the anterior abdominal wall that hinder the increase of intra-abdominal pressure which could be the cause of cryptorchidism in this syndrome.
